# Caffeine Consumption Patterns Among Medical Students: Survey Study

**DOI:** 10.2196/79077

**Published:** 2026-01-29

**Authors:** Brenton Phung, Jonathan Shaw, Seung Rim Yoo, Ashley Lai, Archana Rao, Brian Nguyen, Eileen Ly, James Hagerty, Ryan Chen, Deborah Wright

**Affiliations:** 1 Department of Medical Education, California University of Science and Medicine, 1501 Violet St, Colton, CA, 92324, United States, 1 909-580-9661

**Keywords:** medical education, stress, caffeine, cross-sectional survey, student

## Abstract

This cross-sectional survey of a California medical school found that caffeine consumption increases across medical training, with third-year students consuming more caffeine, particularly from coffee, energy drinks, and over-the-counter stimulants, than first- and second-year students, and higher intake being associated with elevated modified CAGE scores, suggesting stress-related stimulant use.

## Introduction

Medical students frequently use caffeine to maintain alertness, particularly during periods of high academic demand. Prior research shows that nearly 90% of adults in the United States consume caffeine regularly, with elevated use reported among students in rigorous training programs [1-3]. However, little is known about the patterns and potentially problematic caffeine-related behaviors across different stages of medical training. This study assessed caffeine intake and exploratory indicators of problematic caffeine behaviors among first-, second-, and third-year medical students using a cross-sectional design.

## Methods

### Study Design and Participants

A cross-sectional survey was administered to first-year medical students (M1), second-year medical students (M2), and third-year medical students (M3) at a single California medical school in 2024. An email was sent to all these students, and 121 students among all three classes opted to complete the survey. The inclusion criteria were that the participant was to be 18 years of age or older and to be able to provide consent for participation. All 121 participants met the criteria, and no responses were excluded. The final sample included 54 M1s, 45 M2s, and 22 M3s.

### Ethical Considerations

This study received ethical approval from the California University of Science and Medicine Institutional Review Board (approval: HS-2024‐51) on July 11, 2024. Informed consent for primary data collection and secondary analyses of the research data was obtained from all individual participants included in the study. Participants were entered into a raffle where they could win one of twenty US $20 gift cards. Participant privacy was protected by de-identiying all information obtained by preventing the collection of direct identifiers such as email addresses and instead using a study ID for the raffle.

### Survey Instrument

The survey collected demographic characteristics and self-reported use of coffee, tea, energy drinks, sodas, and over-the-counter caffeine preparations (Multimedia Appendix 1). Participants were asked about only standardized serving sizes and not how much caffeine each serving contained.

To explore maladaptive caffeine-related behaviors, we adapted the four-item CAGE questionnaire to caffeine use. This tool has not been validated for caffeine; therefore, it was used only for exploratory screening.

### Outcome Measures

The primary outcomes included (1) the daily caffeine intake (mg/day), (2) the proportion of participants consuming ≥400 mg/day, a widely accepted upper safe limit for adults [4]; and (3) the endorsement of ≥1 adapted CAGE item that was obtained using the descriptive data only.

### Statistical Analysis

Descriptive statistics are reported as mean (SD) or median (IQR). Group differences across training years were evaluated using ANOVA or Kruskal-Wallis tests as appropriate. Analyses were descriptive and not adjusted for confounding variables such as sleep, stress, or baseline stimulant use.

## Results

### Participant Characteristics

Table 1 presents the sample characteristics. The cohort included 61.2% (74/121) female medical students, with a mean age of 24.1 (SD 1.7) years.

**Table:** Table 1. Demographics and reported caffeinated substance intake of 121 medical students in 2025.

Demographics	First-year medical students	Second-year medical students	Third-year medical students	Male students	Female students
Which school year are you? n (%)	54 (44.6)	45 (37.2)	22 (18.2)	—^a^	—
What gender do you identify as? n (%)	—	—	—	47 (38.8)	74 (61.2)
Weekly servings of caffeine products^b^, mean (SD)					
Coffee	5.8 (4.93)	5.36 (4.94)	8.67 (4.13)	6.8 (5.8)	5.7 (4.31)
Tea	3.04 (4.1)	3 (3.91)	2.67 (3.57)	2.78 (3.83)	3.07 (3.98)
Soft drinks	1.92 (4.27)	1.33 (2.81)	0.72 (1.02)	1.95 (4.04)	1.23 (2.93)
Chocolate	2.02 (2.51)	1.24 (1.41)	3 (4.63)	1.53 (2.18)	2.09 (2.96)
Energy drinks	0.61 (1.81)	0.74 (1.67)	0.61 (1.09)	0.75 (2.01)	0.61 (1.41)
Over-the-counter caffeine	0.06 (0.43)	0.02 (0.15)	0.11 (0.32)	0 (0)	0.09 (0.41)
Weekly caffeine consumption (mg), mean (SD)					
Coffee	421.7 (378.6)	398 (388.2)	635.2 (313.2)	495.9 (446.9)	420 (333.4)
Tea	139.7 (192.9)	154.9 (213.1)	122.3 (171.6)	134.8 (190.7)	147.3 (200.7)
Soft drinks	77.9 (181.2)	54.3 (119.3)	29.9 (42.4)	81.8 (179)	48.8 (118)
Chocolate	40.5 (57.9)	29.7 (45.3)	76.3 (119.1)	35.8 (55.6)	46 (75.8)
Energy drinks	96 (294.1)	100.7 (232.7)	98.1 (179.3)	118.3 (323.7)	86.5 (203.1)
Over-the-Counter caffeine	3.98 (27.86)	4.76 (30.86)	7.22 (21.02)	0 (0)	7.61 (34.83)
Total caffeine	779.9 (756)	742.4 (492.4)	968.9 (493.8)	866.5 (854.8)	756.2 (443.7)
Modified CAGE^c^, mean (SD)					
Have you ever felt you needed to cut down on your caffeine intake?	0.45 (0.5)	0.29 (0.46)	0.39 (0.5)	0.45 (0.5)	0.33 (0.48)
Have people annoyed you by criticizing your caffeine intake?	0.18 (0.39)	0.14 (0.35)	0.11 (0.32)	0.13 (0.34)	0.17 (0.38)
Have you ever felt guilty about your caffeine intake?	0.2 (0.41)	0.19 (0.4)	0.11 (0.32)	0.2 (0.41)	0.17 (0.38)
Have you ever felt you needed to consume caffeine first thing in the morning to steady your nerves or to get rid of a headache?	0.31 (0.47)	0.31 (0.47)	0.5 (0.51)	0.23 (0.42)	0.41 (0.5)
Total modified CAGE score	1.14 (1.26)	0.93 (1.18)	1.11 (1.08)	1 (1.09)	1.09 (1.26)

a not applicable.

b The caffeine content per serving for each individual item and its source can be seen in Multimedia Appendix 2.

c The modified CAGE is a proprietary version of the CAGE questionnaire used in studying alcohol use, but has been modified to replace alcohol with caffeine in each question.

### Daily Caffeine Intake

The mean daily caffeine intake was 114.3 (SD 625.7) mg/day. Intake generally increased by training year (M1: 111.4 mg/day; M2: 106.1 mg/day; M3: 138.4 mg/day), although the differences were not statistically significant (*P*=.09). 1/121 of participants consumed ≥400 mg/day of caffeine.

### Caffeine Sources

Coffee was the primary source of caffeine (96/121), followed by tea (85/121), sodas (51/121), and energy drinks (33/121). Over-the-counter caffeine products were used by 5/121.

### Adapted CAGE Indicators

63/121 endorsed ≥1 adapted CAGE item. The most common was “felt the need to cut down” (42/121 or 35%). Because the CAGE-caffeine tool is unvalidated, these findings are interpreted only as exploratory descriptors and not diagnostic indicators.

## Discussion

### Principal Findings

In this single-institution cross-sectional study, medical students reported moderate caffeine intake, with one student exceeding the daily upper limit of 400 mg recommended for adults. Caffeine intake increased across training years, although the differences did not reach statistical significance. These findings are consistent with prior research showing rising caffeine use with progressing academic demands [3].

### Limitations

A key limitation is the lack of validated screening tools for problematic caffeine use. Our adapted CAGE-caffeine items were included only to explore behavioral tendencies; they cannot indicate caffeine use disorder or dependence. Future work should employ validated caffeine behavior instruments or include psychometric testing. Other limitations include potential recall bias, the absence of important confounders such as sleep and stress, restricted generalizability due to the single-site sample, and potential self-selection bias. The potential for winning gift cards could have potentially attenuated some self-selection bias by providing a generalized incentive for participation. Despite these limitations, the study provides descriptive data on caffeine use patterns in medical training and highlights the need for validated tools to assess maladaptive caffeine behaviors.

### Conclusion

Medical students commonly consume caffeine, with a minority exceeding recommended daily limits. Exploratory findings suggest some students may wish to reduce intake. Future studies should incorporate validated instruments and longitudinal designs to better evaluate changes over time.

## Supplementary material

10.2196/79077 Multimedia Appendix 1 Survey used in study containing questions asking about caffeinated substance usage and the modified CAGE questions.

10.2196/79077 Multimedia Appendix 2 Documentation of caffeine values used in data analysis.

## Abbreviations

M1: first-year medical student
M2: second-year medical student
M3: third-year medical student
