# Supplementary material for: Caffeine Consumption Patterns Among Medical Students: Survey Study
Source: JMIR Form Res. 2026 Jan 29;10:e79077. doi: 10.2196/79077 (PMC12854690; doi:10.2196/79077)
Supplement: Multimedia Appendix 1 [file formative-v10-e79077-s001.pdf]

Changes in Caffeine Usage

Changes in Caffeine Usage

PURPOSE:
You are invited to participate in a study examining caffeine usage of the current M1, M2, and M3 cohort at CUSM. This data is meant to quantify the amount of caffeine usage and changes in that usage within students at CUSM.

VOLUNTARY PARTICIPATION: Please understand that participation is completely voluntary. You have the right to withdraw from the study at any time. You also have the right to refuse to answer any question(s) for any reason. If you decide to participate in this study, you will be asked to answer questions about your current eating habits. The answers you provide are very important to us as they help us understand more about the nutritional quality of current first and second year medical students. The study will take approximately 5 to 10 minutes to complete.

CONFIDENTIALITY: Any information that is obtained from you in connection to this study will remain confidential and will be kept confidential to the extent allowed by law. If any data obtained is used for future studies, the anonymity of your responses will be ensured. For the purposes of longitudinal tracking, a unique ID will be created and used only within this study. Anonymized results of the study will be shared with relevant institutional organizations/individuals for the purpose of improving student wellness.

REQUIREMENTS FOR PARTICIPATION: You must be a current first, second, or third year MD student at California University of Science and Medicine who is 18 years or older to participate.

RISKS & BENEFITS: This study does not present more than a minimal risk to participants. There are no foreseeable risks of this study beyond the normal risks of completing a survey. If at any time you are not comfortable answering a question, you may skip it or withdraw from the study with no consequence. Participants will not be compensated for participating in this study. Additionally, the results of this study will be provided to the CUSM faculty and administration, so participants stand to benefit from possibly increased spending for mental health resources and/or events that are meant to improve student mental health.

COMPENSATION: Participants will not be compensated for participating in this study. Moreover, your decision as to whether or not to complete this study will not prejudice your future relationship with CUSM or anyone involved with this study.

Should you have any further questions, please contact Brenton Phung (brenton.phung@md.cusm.edu). You must be at least 18 years old to participate. By agreeing to participate in this study, you understand

the above information and have had all of your questions about participation in this research project answered.

1. By agreeing to participate in this study, you understand the above information and have had all of your questions about participation in this study answered. Do you consent to participate in this study?

   Mark only one oval.

   - Yes - I consent *Skip to question 2*
   - No - I do not consent *Skip to section 3 (Participation Declined)*

Participation Accepted

Thank you for consenting to participating in our study. Should you have any further questions, please contact Brenton Phung (brenton.phung@md.cusm.edu).

2. Which school year are you?

   Mark only one oval.

   - M1 (Class of 2028)
   - M2 (Class of 2027)
   - M3 (Class of 2026)

3. ID Creation: Please create an ID using the number designation of your birth month (Ex. January = 01) combined with the last two digits of your phone number.

   ____

4. What gender do you identify as?

Mark only one oval.

- ( ) Male
- ( ) Female
- ( ) Other: ____________________

5. Do you drink coffee?

Mark only one oval.

- ( ) Yes
- ( ) No

6. How many standard servings of coffee did you consume per week within the last month?

Mark only one oval per row.

	0	1	2	3	4	5	6	7
**Instant Coffee (1 serving = 1 teaspoon)**	◯	◯	◯	◯	◯	◯	◯	◯
**Plunger/ Drip Coffee (1 serving = 6 fl. oz.)**	◯	◯	◯	◯	◯	◯	◯	◯
**Espresso Coffee (such as lattes or americanos) (1 serving = 1 shot of espresso = 1 fl. oz)**	◯	◯	◯	◯	◯	◯	◯	◯
**Cold Brew (1 serving = 6 fl. oz)**	◯	◯	◯	◯	◯	◯	◯	◯

7. What roast level of coffee do you usually drink?

Mark only one oval.

- ◯ Light Roast
- ◯ Medium Roast
- ◯ Dark Roast
- ◯ No preference

8. Do you drink tea?

Mark only one oval.

- Yes
- No

9. How many standard servings of tea did you consume per week within the last month?

Mark only one oval per row.

	0	1	2	3	4	5	6	7	8+
**Green Tea (1 serving = 8 fl. oz)**									
**Black Tea (1 serving = 8 fl. oz)**									
**Cold Brew (1 serving = 8 fl. oz)**									
**Matcha (1 serving = 8 fl. oz)**									

10. Do you drink caffeinated soft drinks?

Mark only one oval.

- Yes
- No

11. How many standard servings of caffeinated soft drinks did you consume per week within the last month?

Mark only one oval per row.

	0	1	2	3	4	5	6	7	[illegible]
**Coca-Cola**									
**Diet Coke**									
**Pepsi**									
**Diet Pepsi**									
**Mountain Dew**									
**Dr Pepper**									
**Diet Dr Pepper**									

12. Do you eat chocolate?

Mark only one oval.

- Yes
- No

13. How many standard servings of chocolate products did you consume per week within the last month?

Mark only one oval per row.

	0	1	2	3	4	5	6	7
**Milk Chocolate (1 serving = 1 bar = 1.5 - 2.0 oz)**	⬭	⬭	⬭	⬭	⬭	⬭	⬭	⬭
**Dark Chocolate (1 serving = 1 bar = 1.5 - 2.0 oz)**	⬭	⬭	⬭	⬭	⬭	⬭	⬭	⬭
**Hot Chocolate (1 serving = 1 cup)**	⬭	⬭	⬭	⬭	⬭	⬭	⬭	⬭

14. Do you take caffeinated over-the-counter drugs?

Mark only one oval.

⬭ Yes

⬭ No

15. How many doses of the following OTC drugs did you consume per week within the last month?

Mark only one oval per row.

	0	1	2	3	4	5	6	7	[illegible]
**Vivarin**									
**NoDoz**									
**Excedrin**									
**Vanquish**									
**Anacin**									
**Dristan**									
**Dexatrim**									

16. Do you consume energy drinks? (Red Bull, V, Mother, Monster Energy, Celsius, Yerba Mate, etc.)

Mark only one oval.

- Yes
- No

17. How many standard servings of energy drinks did you consume per week within the last month?

Mark only one oval per row.

	0	1	2	3	4	5	6	7	8
**Monster Energy**									
**Red Bull**									
**5-Hour Energy**									
**Celsius**									
**Yerba Mate**									
**V**									
**Mother**									

18. Have you ever felt you needed to cut down on your caffeine intake?

Mark only one oval.

- Yes
- No

19. Have people annoyed you by criticizing your caffeine intake?

Mark only one oval.

- Yes
- No

20. Have you ever felt guilty about your caffeine intake?

Mark only one oval.

- Yes
- No

21. Have you ever felt you needed to consume caffeine first thing in the morning to steady your nerves or to get rid of a headache?

Mark only one oval.

- Yes
- No

Participation Declined

You have chosen to not participate in this survey. You can choose to submit your declination or to exit the tab on your browser. Thank you for your time.

This content is neither created nor endorsed by Google.

Google Forms
