# Supplementary material for: Caffeine Consumption Patterns Among Medical Students: Survey Study
Source: JMIR Form Res. 2026 Jan 29;10:e79077. doi: 10.2196/79077 (PMC12854690; doi:10.2196/79077)
Supplement: Multimedia Appendix 2 [file formative-v10-e79077-s002.docx]

### **Multimedia Appendix 2**

### **1) Coffee**

| **Type of Coffee** | **Serving Size** | **Approx. Caffeine Content** | **Source** |
| --- | --- | --- | --- |
| **Espresso** | 1 fl oz (30 mL) | 63 mg | https://www.mayoclinic.org/healthy-lifestyle/nutrition-and-healthy-eating/in-depth/caffeine/art-20049372 |
| **Instant Coffee** | 1 teaspoon (~1.8 g)  * One teaspoon (~1.8 grams) of instant coffee typically makes 1 standard cup (8 fl oz or ~240 mL) of coffee. | ~60 mg | https://nutritionsource.hsph.harvard.edu/caffeine/ |
| **Plunger/Drip Coffee** | 6 fl oz (177 mL) | ~ 98 mg | Cappelletti, S., Piacentino, D., Sani, G., & Aromatario, M. (2015). Caffeine: cognitive and physical performance enhancer or psychoactive drug?. Current neuropharmacology, 13(1), 71–88. https://doi.org/10.2174/1570159X13666141210215655 |
| **Cold Brew Coffee** | 6 fl oz (177 mL) | 72 mg | https://www.mayoclinic.org/healthy-lifestyle/nutrition-and-healthy-eating/in-depth/caffeine/art-20049372 |

### **2) Tea**

| **Type of Tea** | **Serving Size** | **Approx. Caffeine Content** | **Source** |
| --- | --- | --- | --- |
| **Black Tea** | 8 fl oz (237 mL) | 48 | https://www.mayoclinic.org/healthy-lifestyle/nutrition-and-healthy-eating/in-depth/caffeine/art-20049372 |
| **Green Tea** | 8 fl oz (237 mL) | 29 | https://www.mayoclinic.org/healthy-lifestyle/nutrition-and-healthy-eating/in-depth/caffeine/art-20049372 |
| **Matcha** | 8 fl oz (237 mL) | ~72.5 mg | [Koláčková et al., 2021](https://doi.org/10.3390/molecules26010085) |

### **3) Energy Drinks**

| **Brand** | **Serving Size** | **Caffeine Content** | **Source** |
| --- | --- | --- | --- |
| **Monster Energy** | 16 fl oz (473 mL) | 160 mg | Nutrition |
| **Red Bull** | 8.4 fl oz (250 mL) | 80 mg | Nutrition |
| **5-Hour Energy** | 2 fl oz (60 mL) | 200 mg | Nutrition |
| **Celsius** | 12 fl oz (355 mL) | 200 mg | Nutrition |
| **Yerba Mate** | 8 fl oz (237 mL) | ~85 mg | Nutrition |
| **V Energy Drink** | 8.3 fl oz (250 mL) | 80 mg | Nutrition |
| **Mother Energy** | 16 fl oz (500 mL) | 160 mg | Nutrition |

* The CaffCo (Caffeine Consumption Habits Questionnaire) does not specify a universal standard serving size for energy drinks. Instead, it assesses caffeine intake by collecting detailed information on the specific products consumed, their portion sizes, and consumption frequency.

### **4) Soda (12 oz / 355 mL)**

| **Brand** | **Caffeine Content** | **Source** |
| --- | --- | --- |
| **Coca-Cola** | 34 mg | Nutrition |
| **Diet Coke** | 46 mg | Nutrition |
| **Pepsi** | 38 mg | Nutrition |
| **Diet Pepsi** | 35 mg | Nutrition |
| **Mountain Dew** | 54 mg | Nutrition |
| **Dr. Pepper** | 41 mg | Nutrition |
| **Diet Dr. Pepper** | 41 mg | Nutrition |

### **5) Chocolate**

| **Type of Chocolate** | **Serving Size** | **Approx. Caffeine Content** | **Source** |
| --- | --- | --- | --- |
| **Milk Chocolate** | 1.5–2.0 oz (43–57 g) | ~9–12 mg | https://nutritionsource.hsph.harvard.edu/caffeine/ |
| **Dark Chocolate (70–85% cacao)** | 1.5–2.0 oz (43–57 g) | 36-48 mg | https://nutritionsource.hsph.harvard.edu/caffeine/ |
| **Hot Chocolate** | 1 cup (8 fl oz) | ~6 mg | Barone, J. J., & Roberts, H. R. (1996). Caffeine consumption. Food and chemical toxicology : an international journal published for the British Industrial Biological Research Association, 34(1), 119–129. https://doi.org/10.1016/0278-6915(95)00093-3 |

### **6) Over-the-Counter (OTC) Drugs**

| **Product** | **Caffeine Content per Pill** | **Source** |
| --- | --- | --- |
| **Vivarin** | 200 mg | Nutrition |
| **NoDoz** | 200 mg | Nutrition |
| **Excedrin** | 65 mg | Nutrition |
| **Vanquish** | 33 mg | Nutrition |
| **Anacin** | 32 mg | Nutrition |
| **Dristan** | 16 mg | Nutrition |
| **Dexatrim** | 200 mg | Nutrition |

**Reference List**

1. Mayo Clinic Staff. Caffeine: How much is too much? Mayo Clinic. Updated March 6, 2024. Accessed January 28, 2026. https://www.mayoclinic.org/healthy-lifestyle/nutrition-and-healthy-eating/in-depth/caffeine/art-20049372
2. Harvard T.H. Chan School of Public Health. Caffeine. The Nutrition Source. Accessed January 28, 2026. https://nutritionsource.hsph.harvard.edu/caffeine/
3. Cappelletti S, Piacentino D, Sani G, Aromatario M. Caffeine: cognitive and physical performance enhancer or psychoactive drug? Curr Neuropharmacol. 2015;13(1):71-88. doi:10.2174/1570159X13666141210215655
4. Kochman J, Jakubczyk K, Antoniewicz J, Mruk H, Janda K. Health benefits and chemical composition of matcha green tea: a review. Molecules. 2021;26(1):85. doi:10.3390/molecules26010085
5. Monster Energy. Monster Energy Original Green. Accessed January 28, 2026. https://www.monsterenergy.com/en-gb/energy-drinks/monster-energy/original-green/
6. Red Bull. How much caffeine is in a can of Red Bull Energy Drink? RedBull.com. Accessed January 28, 2026. https://www.redbull.com/us-en/energydrink/questions/how-much-caffeine-is-in-a-can-of-red-bull-energy-drink
7. 5-hour ENERGY. 5-hour ENERGY Caffeine Facts. 5hourenergy.com. Accessed January 28, 2026. https://5hourenergy.com/blogs/the-feed/5-hour-energy-caffeine-facts
8. Celsius. Essential Facts. Celsius.com. Accessed January 28, 2026. https://www.celsius.com/essential-facts/
9. Yerba Madre. Enlighten Mint Product Page. Yerbamadre.com. Accessed January 28, 2026. https://yerbamadre.com/products/enlightenmint
10. Caffeine Informer. Caffeine Content — V. CaffeineInformer.com. Accessed January 28, 2026. https://www.caffeineinformer.com/caffeine-content/v
11. Caffeine Informer. Caffeine Content — Mother New. CaffeineInformer.com. Accessed January 28, 2026. <https://www.caffeineinformer.com/caffeine-content/mother-new>
12. The Coca-Cola Company. How much caffeine is in a Coca-Cola? Coke.com. Accessed January 28, 2026. https://www.coca-cola.com/xe/en/about-us/faq/how-much-caffeine-is-in-a-cocacola
13. The Coca-Cola Company. What is caffeine? Coke.com. Accessed January 28, 2026. https://www.coca-cola.com/us/en/about-us/faq/what-is-caffeine
14. PepsiCo. Caffeine content — product facts. PepsiCoProductFacts.com. Accessed January 28, 2026. https://www.pepsicoproductfacts.com/home/caffeine
15. Keurig Dr Pepper. Dr Pepper 16 fl oz (US) — Product Facts. KDPProductFacts.com. Accessed January 28, 2026. https://www.kdpproductfacts.com/product/a0e3h000003LJzcAAG/dr-pepper-16-fl-oz-us
16. Keurig Dr Pepper. Diet Dr Pepper 12 fl oz (US) — Product Facts. KDPProductFacts.com. Accessed January 28, 2026. https://www.kdpproductfacts.com/product/a0e3h000003LK0AAAW/diet-dr-pepper-12-fl-oz-us
17. Barone JJ, Roberts HR. Caffeine consumption. Food Chem Toxicol. 1996;34(1):119-129. doi:10.1016/0278-6915(95)00093-3
18. Vivarin. Vivarin caffeine tablets. Vivarin.com. Accessed January 28, 2026. https://vivarin.com/products/vivarin?srsltid=AfmBOopYfreNSFoY2DukVfF3RKljwX2ZOFsmLbJXAixUlLtPDoEhJ87R
19. NoDoz. Good, clean energy — NoDoz caffeine pills. NoDoz.com. Accessed January 28, 2026. https://www.nodoz.com/
20. Excedrin. Extra Strength Head Pain Relief caplets. Excedrin.com. Accessed January 28, 2026. https://www.excedrin.com/products/head-pain-relief/extra-strength-caplets/
21. DailyMed. EXCEDRIN EXTRA STRENGTH (acetaminophen, aspirin, and caffeine) — Drug labeling. DailyMed.nlm.nih.gov. Accessed January 28, 2026. https://dailymed.nlm.nih.gov/dailymed/fda/fdaDrugXsl.cfm?setid=f879e8c7-1983-4a85-b87d-38f5068351fc&type=display
22. Anacin. Anacin Advantage aspirin + caffeine headache relief products. Anacin.com. Accessed January 28, 2026. https://www.anacin.com/anacin-advantage-aspirin-caffeine-headache-relief-products
23. Health Canada DHPP. Product information document (42083). dhpp.hpfb-dgpsa.ca. Accessed January 28, 2026. https://dhpp.hpfb-dgpsa.ca/dhpp/resource/42083
24. Dexatrim.com. Dexatrim dietary supplement website. Accessed January 28, 2026. http://www.dexatrim.com/
